# Somatostatin receptor 2A in gliomas: Association with oligodendrogliomas and favourable outcome

**DOI:** 10.18632/oncotarget.17097

**Published:** 2017-04-13

**Authors:** Aida Kiviniemi, Maria Gardberg, Katri Kivinen, Jussi P. Posti, Ville Vuorinen, Jussi Sipilä, Melissa Rahi, Matti Sankinen, Heikki Minn

**Affiliations:** ^1^ Department of Radiology, Medical Imaging Center of Southwest Finland, Turku University Hospital and University of Turku, Turku, Finland; ^2^ Turku PET Center, Turku University Hospital and University of Turku, Turku, Finland; ^3^ Department of Pathology, Turku University Hospital and University of Turku, Turku, Finland; ^4^ TYKSLAB, Laboratory of Molecular Genetics, Turku University Hospital, Turku, Finland; ^5^ Division of Clinical Neurosciences, Department of Neurosurgery, Turku University Hospital and University of Turku, Turku, Finland; ^6^ Department of Neurology, North Karelia Central Hospital, Joensuu, Finland; ^7^ Division of Clinical Neurosciences, Department of Neurology, Turku University Hospital and University of Turku, Turku, Finland; ^8^ Department of Oncology and Radiotherapy, Turku University Hospital, Turku, Finland

**Keywords:** glioma, somatostatin receptor, oligodendroglioma, IDH mutation, prognosis

## Abstract

Somatostatin receptor subtype 2A (SSTR2A) is a potential therapeutic target in gliomas. Data on SSTR2A expression in different glioma entities, however, is particularly conflicting. Our objective was to characterize SSTR2A status and explore its impact on survival in gliomas classified according to the specific molecular signatures of the updated WHO classification. In total, 184 glioma samples were retrospectively analyzed for SSTR2A expression using immunohistochemistry with monoclonal antibody UMB-1. Double staining with CD68 was used to exclude microglia and macrophages from analyses. SSTR2A staining intensity and its localization in tumor cells was evaluated and correlated with glioma entities and survival. Diagnoses included 101 glioblastomas (93 *isocitrate dehydrogenase (IDH)* -wildtype, 3 *IDH*-mutant, 5 not otherwise specified (NOS)), 60 astrocytomas (22 *IDH*-wildtype, 37 *IDH*-mutant, 1 NOS), and 23 oligodendrogliomas (19 *IDH*-mutant and 1p/19q-codeleted, 4 NOS). SSTR2A expression significantly associated with oligodendrogliomas (79% SSTR2A positive) compared to *IDH*-mutant or *IDH*-wildtype astrocytomas (27% and 23% SSTR2A positive, respectively), and especially glioblastomas of which only 13% were SSTR2A positive (*p* < 0.001, Fisher's exact test). The staining pattern in glioblastomas was patchy whereas more homogeneous membranous and cytoplasmic staining was detected in oligodendrogliomas. Positive SSTR2A was related to longer overall survival in grade II and III gliomas (HR 2.7, CI 1.2–5.8, *p* = 0.013). In conclusion, SSTR2A expression is infrequent in astrocytomas and negative in the majority of glioblastomas where it is of no prognostic significance. In contrast, oligodendrogliomas show intense membranous and cytoplasmic SSTR2A expression, which carries potential diagnostic, prognostic, and therapeutic value.

## INTRODUCTION

Gliomas are the most common malignant primary brain tumors [1]. Despite optimal standard of care including maximal safe resection followed by radiotherapy and/or chemotherapy, the median survival in glioblastomas (WHO grade IV) is only 15 months [2]. Grade II gliomas are slow-growing but have an intrinsic tendency over time for malignant transformation resulting in a median survival of approximately ten years [3]. This limited response to standard therapies is related to the infiltration of glioma cells into the surrounding “normal” brain, which shelters them from surgery and radiation [4].

Updated WHO classification of central nervous system tumors combines for the first time histological and molecular features for an integrated classification [5]. *IDH* mutation is the key genetic feature characterizing grade II and III gliomas as well as secondary glioblastomas with favourable outcome [6]. Furthermore, oligodendrogliomas are recognized by their expression of two major genetic alterations, *IDH* mutation and 1p/19q codeletion.

SSTRs are a family of G protein-coupled receptors consisting of six different subtypes (SSTR1, 2A, 2B, 3, 4 and 5). Various solid tumors express SSTRs with the potential of somatostatin analogs to exert anti-tumor effects [7]. SSTR2A is the most abundant subtype and is used in neuroendocrine tumors (NETs) as a target for both diagnostic positron emission tomography/computed tomography (PET/CT) imaging and radionuclide therapy using somatostatin analogs labeled with β^−^-emitting isotopes (^90^Y-DOTATOC and ^177^Lu-DOTATATE) [8, 9].

SSTR2A targeted radionuclide therapy has been suggested as a novel treatment approach in gliomas [10–12]. Tumor SSTR2A expression is required for such an approach to be successful, however, characterization of this target in gliomas has thus far been controversial. Studies with limited number of patients have reported high SSTR2 expression in glioblastomas and low expression in grade II–III gliomas [13, 14], while others have detected the opposite [15].

We have recently found that PET/CT imaging targeting SSTR2A with ^68^Ga-labeled DOTA-chelated peptides in high-grade gliomas does not correlate with SSTR2A immunohistochemistry suggesting that imaging may not act as a surrogate marker for receptor expression [16]. A finding of particular interest in this pilot evaluation of 28 patients was the association between SSTR2A expression and oligodendroglial component, *IDH1* mutation, and progression-free survival (PFS).

This potentially interesting prognostic information led us to more extensively characterize SSTR2A expression in glioma entities using the specific molecular signatures of the updated 2016 WHO classification. Our objective was to examine the impact of SSTR2A status on survival across 184 cases of gliomas representing different molecular and histological features. We hypothesized that SSTR2A status in gliomas may provide a diagnostic and prognostic tool useful for therapeutic decision-making and predicting the outcome.

## RESULTS

### Patient cohort

A total number of 184 gliomas were included in this retrospective study with 101 glioblastomas (93 *IDH*-wildtype, 3 *IDH*-mutant, 5 NOS), 60 astrocytomas (22 *IDH*-wildtype, 37 *IDH*-mutant, 1 NOS), and 23 oligodendrogliomas (19 *IDH*-mutant and 1p/19q-codeleted, 4 NOS). Basic patient characteristics, clinical follow-up data, alpha-thalassemia/mental retardation syndrome X-linked (*ATRX*) and *p53* mutations within molecular diagnoses are presented in Table 1. Direct sequencing detected one *IDH2* mutation in a grade II astrocytoma and additionally six *IDH1* mutations were identified not attributed to R132H mutation or from samples with missing IDH1 immunohistochemistry. Four oligodendrogliomas with 1p/19q codeletion and typical oligodendroglial histology were designated to NOS group since *IDH1/IDH2* mutation could not be detected.

**Table 1:** Patient characteristics

	GLIOBLASTOMA			ASTROCYTOMA			OLIGODENDROGLIOMA	
	*IDH*-wildtype	*IDH*-mutant	NOS	*IDH*-wildtype	*IDH*-mutant	NOS	*IDH*-mutant and 1p/19q-codeleted	NOS
n	93	3	5	22	37	1	19	4
Median age (range), years	62 (22–79)	58 (53–67)	57 (50–69)	61 (18–79)	39 (21–83)	33	51 (23–69)	48 (27–54)
Grade								
IV	93 (100%)	3 (100%)	5 (100%)	-	-	-	-	-
III	-	-	-	18 (82%)	15 (41%)	-	6 (32%)	-
II	-	-	-	4 (18%)	22 (59%)	1 (100%)	13 (68%)	4 (100%)
Preoperative KPS% (median)	70	70	70	80	90	70	90	90
Resection								
Gross total	20 (22%)	0	0	2 (9%)	10 (27%)	0	1 (5%)	1 (25%)
Subtotal	73 (78%)	3 (100%)	5 (100%)	17 (77%)	26 (70%)	1 (100%)	17 (90%)	3 (75%)
Biopsy	0	0	0	3 (14%)	1 (3%)	0	1 (5%)	0
Postoperative treatment								
None	14 (15%)	0	1 (20%)	4 (18%)	11 (30%)	0	2 (11%)	1 (25%)
RT alone	27 (30%)	0	1 (20%)	16 (73%)	23 (62%)	1 (100%)	16 (84%)	3 (75%)
RT + TMZ	50 (55%)	2 (67%)	3 (60%)	2 (9%)	3 (8%)	0	1 (5%)	0
RT+Sitimagene ceradenovec	0	1 (33%)	0	0	0	0	0	0
*ATRX* mutation								
Yes	0	1 (50%)	0	0	25 (81%)	0	0	0
No	82 (100%)	1 (50%)	4 (100%)	18 (100%)	6 (19%)	0	18 (100%)	1 (100%)
Information missing	11	1	1	4	6	1	1	3
*p53* mutation								
Yes	40 (43%)	3 (100%)	3 (60%)	6 (27%)	29 (78%)	1 (100%)	0	0
No	53 (57%)	0	2 (40%)	16 (73%)	8 (22%)	0	19 (100%)	4 (100%)

Abbreviations: KPS Karnofsky Performance Scale, RT radiotherapy, TMZ temozolomide, Sitimagene ceradenovec (Cerepro^®^) experimental gene therapy.

### Scoring of SSTR2A immunohistochemistry and its association with tumor entity

**Figure 1:**
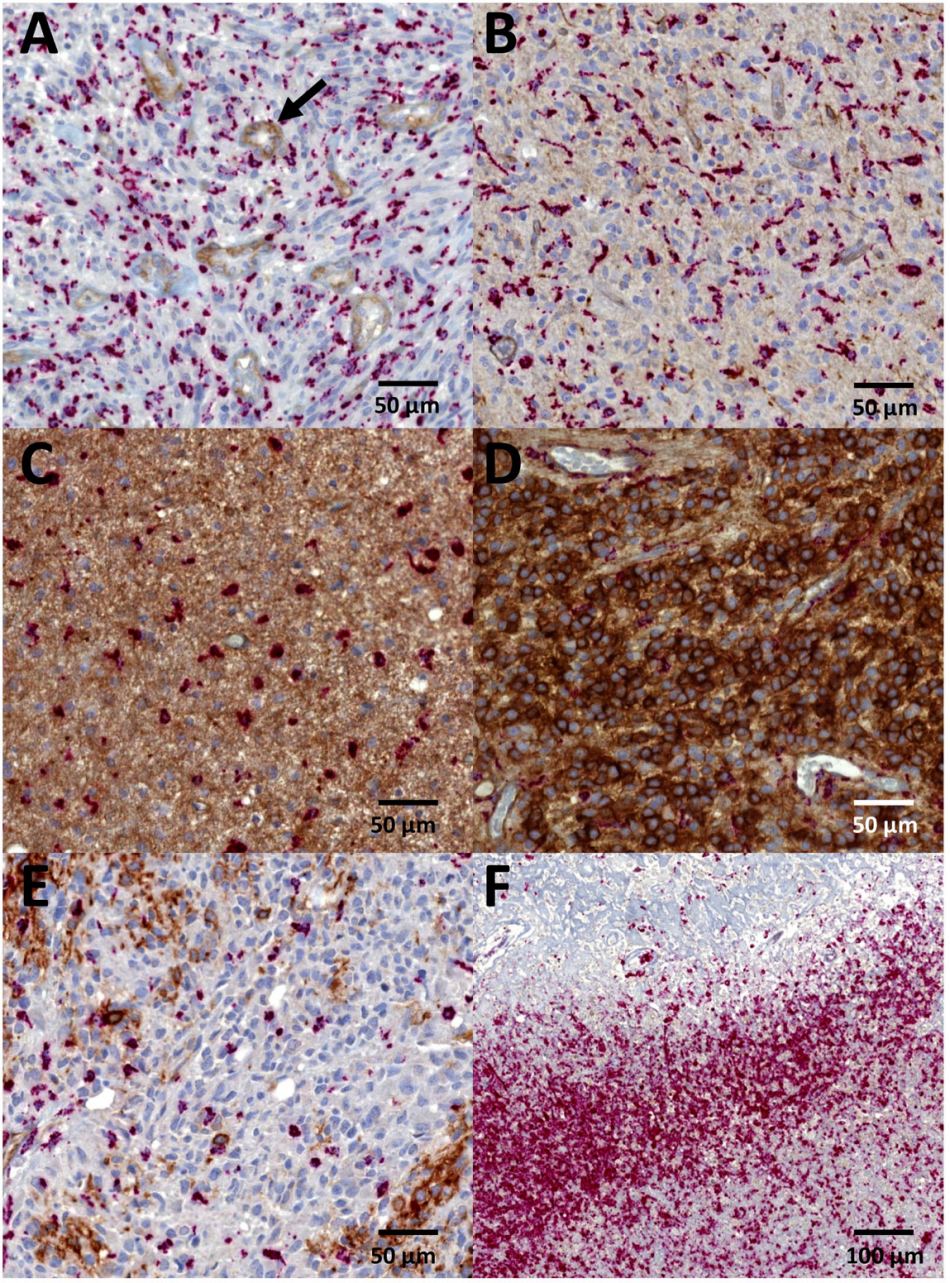
Immunohistochemistry for SSTR2A Intensity of SSTR2A staining was scored (**A**) negative = 0, (**B**) weak = 1, (**C**) moderate = 2, or (**D**) strong = 3. Location was designated as (B, C) cytoplasmic or (D) both membranous and cytoplasmic. Endothelial cells served as internal positive control (black arrow). (**E**) Heterogeneous SSTR2A expression with patchy staining was observed especially in glioblastomas. (**F**) No SSTR2A staining could be detected in CD68 positive macrophages (red stain) bordering necrotic area. Diagnoses of the representative images included (A, B, E, F) glioblastoma, (C) astrocytoma *IDH*-mutant grade II, and (D) oligodendroglioma *IDH*-mutant and 1p/19q-codeleted grade III.

Representative images of different intensities and localization of SSTR2A immunostaining are shown in Figure 1. High SSTR2A expression significantly associated with oligodendrogliomas whereas the majority of glioblastomas were negative for SSTR2A immunostaining (Table 2). SSTR2A expression varied between glioma subtypes evaluated by the most common staining intensity (minimum 50% of tumor area, *p* < 0.001, Fisher's exact test) and also the highest intensity (minimum 10% of tumor area, *p* < 0.001).

**Table 2:** Scoring of SSTR2A immunohistochemistry

	Most common intensity				Highest intensity				Location	
	0	1	2	3	0	1	2	3	C	M + C
Glioblastoma										
*IDH*-wildtype (*n* = 93)	90 (97%)	1 (1%)	1 (1%)	1 (1%)	57 (61%)	15 (16%)	9 (10%)	12 (13%)	26 (70%)	11 (30%)
*IDH*-mutant (*n* = 3)	3 (100%)	0	0	0	0	2 (67%)	1 (33%)	0	2 (67%)	1 (33%)
Astrocytoma										
*IDH*-wildtype (*n* = 22)	19 (86%)	2 (9%)	1 (5%)	0	12 (54%)	2 (9%)	3 (14%)	5 (23%)	10 (100%)	0
*IDH*-mutant (*n* = 37)	24 (65%)	5 (13.5%)	5 (13.5%)	3 (8%)	9 (24%)	9 (24%)	10 (27%)	9 (24%)	24 (86%)	4 (14%)
Oligodendroglioma										
*IDH*-mutant and 1p/19q-codeleted (*n* = 19)	4 (21%)	1 (5%)	7 (37%)	7 (37%)	0	0	4 (21%)	15 (79%)	7 (37%)	12 (63%)

Abbreviations: C cytoplasmic, M membranous.

The most common pattern of SSTR2A staining was predominantly negative in glioblastomas (97%) and *IDH*-wildtype astrocytomas (86%) but also in the majority of *IDH*-mutant astrocytomas (65%). However, we detected also heterogeneous staining within the negative tumor bulk showing tumor cell clusters with intensive SSTR2A staining. Therefore, we scored the tumor as SSTR2A positive if the most common intensity was 2 or 3, or if the highest intensity was 3. This translated to a positive SSTR2A status in 12 *IDH*-wildtype glioblastomas (13%), 5 *IDH*-wildtype astrocytomas (23%), 10 *IDH*-mutant astrocytomas (27%), and 15 *IDH*-mutant and 1p/19q-codeleted oligodendrogliomas (79%) demonstrating a significant association between SSTR2A status (positive or negative) and glioma type (*p* < 0.001, Fisher's exact test). Accordingly, SSTR2A expression was related to *IDH* mutation since the majority (60%) of SSTR2A positive gliomas harboured *IDH* mutation (*p* < 0.001, Fisher's exact test). However, no association between SSTR2A expression and *ATRX* mutation was observed (*p* = 0.469). Furthermore, within grade II and III gliomas, no difference in SSTR2A status was detected with regard to tumor grade (*p* = 0.485).

In glioblastomas and astrocytomas the SSTR2A expression was mainly located in cytoplasm, whereas in the majority of oligodendrogliomas, the staining pattern was both membranous and cytoplasmic (Table 2, *p* < 0.001, Fisher's exact test). Oligodendrogliomas NOS (*n* = 4) were not included in the final analyses, but it is noteworthy that they all presented SSTR2A intensity of 3 as the most common staining. Furthermore, in three cases the staining was mostly membranous following the pattern of 1p/19q-codeleted tumors.

High number of CD68 positive microglia and macrophages were detected in the tumor zone bordering necrosis in glioblastomas, whereas in areas with high number of viable tumor cells, microglia and macrophages were more randomly dispersed. As shown in Figure 1, no SSTR2A immunoreactivity in microglia and macrophages could be detected through the intense red staining of CD68.

### Association of SSTR2A expression with survival

**Figure 2:**
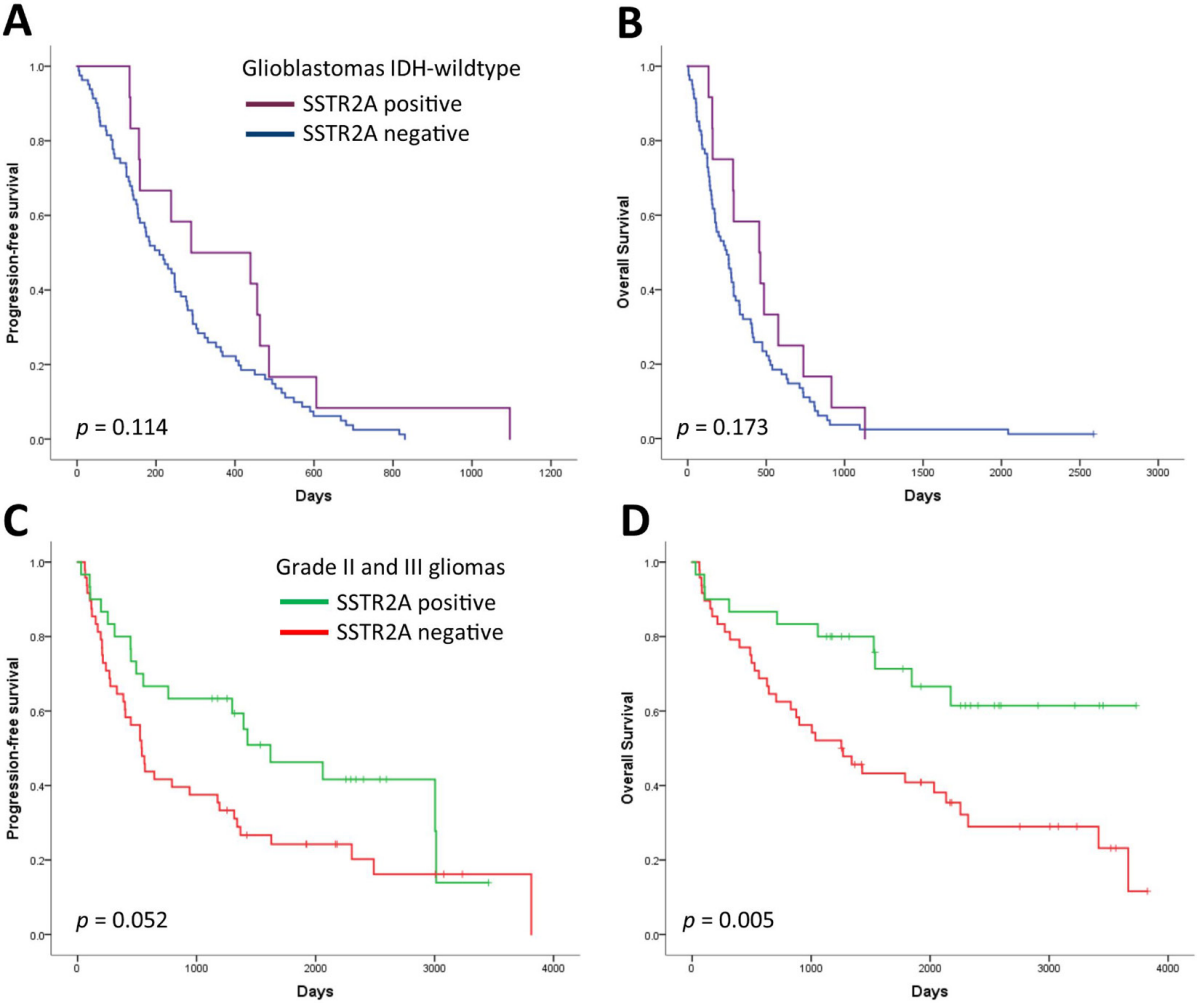
Progression-free survival and overall survival according to SSTR2A status in (**A**, **B**) glioblastomas and (**C**, **D**) grade II–III gliomas.

Survival plots in Figure 2 show no difference in overall survival (OS) or PFS in glioblastomas according to SSTR2A status (*p* = 0.173 and *p* = 0.114, respectively, Log-Rank test). In contrast, patients with SSTR2A positive grade II or III gliomas showed clear survival benefit compared to SSTR2A negative gliomas (OS *p* = 0.005, PFS *p* = 0.052, Log-Rank test). This benefit, however, may be related to the association between SSTR2A and oligodendrogliomas and their favourable outcome since no significant difference in OS (*p* = 0.383) or PFS (*p* = 0.272) was observed within *IDH*-mutant and *IDH*-wildtype astrocytomas according to SSTR2A status.

We also studied in glioblastomas whether membranous SSTR2A expression predicted favourable outcome compared to cytoplasmic expression by scoring SSTR2A positive if tumor cells showed membranous staining. However, no difference in OS (*p* = 0.389) or PFS (*p* = 0.287) was observed.

In the multivariate Cox regression analysis of grade II and III gliomas, both *IDH* mutation (*p* < 0.001) and positive SSTR2A (*p* = 0.013) remained independent factors that were significantly associated with longer overall survival, after adjustment for age, preoperative Karnofsky Performance Scale (KPS), and resection type (Table 3). Since diverging therapies following disease progression may have prognostic relevance, we evaluated whether the treatment at first progression varied between SSTR2A positive and SSTR2A negative grade II and III gliomas (Table 4). No significant difference, however, was observed (*p* = 0.136, Fisher's exact test).

**Table 3:** Multivariate Cox regression model for OS within grade II and III gliomas adjusted for age, Karnofsky performance scale, and resection type

Variable	Hazard ratio	95% CI	*p*
*IDH*-wildtype vs. *IDH*-mutant	5.1	2.4–11.0	< 0.001
SSTR2A negative vs. SSTR2A positive	2.7	1.2–5.8	0.013

**Table 4:** Treatment at first progression in SSTR2A positive and negative grade II–III gliomas (*p* = 0.136, fisher's exact test)

Treatment	SSTR2A positive (*n* = 18)	SSTR2A negative (*n* = 39)
None	4 (22%)	13 (33%)
RT	0	4 (10%)
TMZ	5 (28%)	5 (13%)
Surgery	4 (22%)	10 (26%)
Surgery + RT	0	3 (8%)
Surgery + TMZ	5 (28%)	2 (5%)
Surgery + RT + TMZ	0	1 (2.5%)
Surgery + Lomustine	0	1 (2.5%)

## DISCUSSION

Studies on SSTR2 expression in gliomas have demonstrated remarkably controversial results [13–15]. Our analysis included a total of 184 gliomas and is to our knowledge the most extensive effort to characterize SSTR2 expression in different glioma subtypes assessed by the new 2016 WHO classification system. In our cohort, SSTR2A expression was significantly associated with oligodendrogliomas (79% SSTR2A positive) compared to *IDH*-mutant or *IDH*-wildtype astrocytomas (27% and 23%, respectively) and especially glioblastomas of which only 13% were SSTR2A positive.

In previous studies, Reubi et al. concluded from their autoradiographic assays that SSTRs are predominantly expressed in low-grade and anaplastic gliomas whereas only one out of 20 glioblastomas demonstrated binding of the radiolabeled somatostatin analog *in vitro* [15]. In contrast, another series where 50 tumor samples were assayed with a polyclonal antibody SSTR2A was reported as positive in 44% of glioblastomas, while only 10% of anaplastic and none of diffuse astrocytomas showed positive immunostaining for SSTR2 [13]. Dutour et al. reported expression of SSTR2 mRNA by Northern blot in 6 out of 9 gliomas with the highest expression detected in one glioblastoma and two oligodendrogliomas [14]. Controversial results may be explained by the limitations in the number of tumor samples and especially oligodendrogliomas included, and the analytical methods used in these studies. Autoradiography and Northern blot analyses are unable to define the exact location of the receptor making it impossible to differentiate SSTR2 expression in tumor cells from other cell types such as macrophages which are abundant in gliomas and known to express SSTR2 [17]. Furthermore, immunohistochemistry with polyclonal antibodies may display cross-reactivity with other antigens resulting in false positive staining [18]. We used a monoclonal antibody UMB-1, which is generally recommended as the method of choice for SSTR2A immunohistochemistry due to the more robust staining it provides when compared to polyclonal antisera [19, 20]. Moreover, the interference of SSTR2A expression in macrophages was excluded from analyses by performing double staining with CD68 targeting macrophages and UMB-1 targeting the intracellular C-terminus of SSTR2A.

High SSTR2A expression in oligodendrogliomas carries clinical implications. First, intensive membranous and cytoplasmic SSTR2A staining detected in the majority of tumor cells may add diagnostic value to routine pathologic evaluation since membranous staining was almost exclusively limited to oligodendrogliomas. Four oligodendrogliomas in our cohort with 1p/19q codeletion and typical oligodendroglial histology were designated to NOS group since no *IDH1/IDH2* mutation could be detected. Interestingly, all of these four tumors showed high SSTR2A expression. We hypothesize that intense membranous and cytoplasmic SSTR2A expression could act as a surrogate marker supporting the diagnosis of oligodendroglioma in case of ambiguous or unavailable analysis of 1p/19q or IDH status.

Second clinical implication of SSTR2A expression in gliomas is the therapeutic target it may offer. ^90^Y-DOTATOC and ^177^Lu-DOTATATE are somatostatin analogs, which are labelled with β^−^-emitting isotopes and preferentially bind to SSTR2 [21]. Both ^90^Y-DOTATOC and ^177^Lu-DOTATATE are in clinical use for treating metastatic and inoperable NETs abundantly expressing SSTR2 [8, 22]. Interestingly, recent studies have indicated that SSTR2 antagonists are superior to agonists in targeting tumors, which has emerged new potential indications for SSTR2 targeting even in tumors with low receptor density [23]. With regard to gliomas, encouraging results have been reported in three pilot studies investigating treatment with ^90^Y-DOTATOC in progressive grade II–IV gliomas [10–12]. In contrast to intravenous injection of the radionuclide in NET, a locoregional delivery of ^90^Y-DOTATOC was used in gliomas to circumvent the blood-brain barrier and to allow higher tumor dose while reducing systemic toxicity (especially the kidneys and bone marrow which are the dose-limiting organs in systemic administration). Furthermore, no diffusion into adjacent normal brain areas could be detected on planar cranial scintigrams when administering the radionuclide locally. However, the procedure is technically demanding and clear definition of the patients who are most likely to benefit from it is needed. We have recently demonstrated that PET/CT imaging with intravenously injected ^68^Ga-DOTA-peptide targeting SSTR2 provides limited value in defining suitable patients with high-grade glioma for targeted radionuclide therapy [16]. ^68^Ga-DOTA-peptide uptake was associated with disrupted blood-brain barrier characteristic for glioblastomas, but did not correlate with SSTR2A expression via immunohistochemistry. Similar conclusions were made by Lapa et al. in a coinciding study where 15 glioblastoma samples were analyzed for SSTR2 expression with ^68^Ga-DOTATATE PET/CT performed to three of these patients [24]. Thus, not only receptor expression and density but route of administration may be important for targeted treatment in case of malignant gliomas.

Current study further questions the effectiveness of SSTR2 targeted radionuclide therapy in glioblastomas. We have now characterized SSTR2A expression in 101 glioblastomas and demonstrate completely negative immunostaining for SSTR2 in the majority of tumor samples. In contrast, we found that most oligodendrogliomas show intense SSTR2A expression. Moreover, SSTR2A expression in oligodendrogliomas is mostly localized to the plasma membrane of tumor cells in addition to concurrent cytoplasmic staining. Membranous SSTR2A is known to rapidly internalize after binding of somatostatin analog and this accumulation of internalized radioligand into tumor cells is considered the basis for successful radionuclide therapy [25]. Consequently, the pattern of expression favours theranostic approach using ^90^Y-DOTATOC and ^177^Lu-DOTATATE and might contribute to the treatment armamentarium of oligodendrogliomas, which should be addressed in future clinical trials.

SSTR2 itself is considered to be a tumor suppressor demonstrating significant reduction in pancreatic tumor growth after adenoviral vector-based SSTR2 gene transfer in experimental pancreatic cancer [26]. SSTR2 expression has also been associated with favourable outcome in patients with pancreatic NETs and childhood neuroblastomas [27, 28]. Our study clearly supports similar survival benefit in patients with grade II-III glioma who present with positive SSTR2A status. This may be related to the strong association between SSTR2A expression and oligodendrogliomas which typically demonstrate longer survival times than diffuse astrocytomas. However, the prognostic significance of SSTR2A cannot be trivialized since it remained an independent prognostic factor in the multivariate analysis where *IDH* mutation and clinical determinants were included. Unfortunately, the number of patients in our study was too low to perform a separate survival analysis including oligodendrogliomas only.

Large-scale genomic profiling has defined four subtypes of glioblastomas (proneural, neural, classical, and mesenchymal) beyond IDH mutational status each presenting distinct prognosis and response to therapy [29]. The proneural subtype with beneficial outcome has also been associated with anaplastic oligodendrogliomas with 1p/19q codeletion [30]. Recently, an integrated analysis of transcriptome, genome, and methylome of 156 oligodendroglial tumors identified three subgroups of 1p/19q-codeleted oligodendrogliomas with specific expression patterns and divergent outcomes [31]. In our cohort SSTR2A expression associated with longer OS, however, its relation with the genetic profiles and subtypes of the heterogeneous group of oligodendrogliomas remains to be elucidated.

To our knowledge, this is the most extensive study aiming to characterize SSTR2A expression in adult gliomas. Here we confirm our preliminary observation implicating the association of SSTR2A expression with oligodendroglial differentiation where it may provide diagnostic and therapeutic value complementing the new molecular classification. In contrast, glioblastomas present negative or small patchy areas of SSTR2A staining supporting the observation that glioblastomas are composed of numerous different clones with variable biological properties where theranostic approach using DOTA-labeled peptides has low priority.

## MATERIALS AND METHODS

### Patients and clinical data

A retrospective analysis was performed on adult patients with newly diagnosed supratentorial glioma grade II-IV who underwent surgical resection or biopsy at Turku University Hospital from January 2005 through December 2013. A total of 184 glioma samples were included. Clinical data was collected from the electronic patient data system. The study was approved by the Ethics Committee of the Hospital District of Southwest Finland and Auria Biobank. The samples were obtained from Auria Biobank (TYKS-SAPA, Turku University Hospital, Turku, Finland), and in accordance with the Finnish Biobank Act (688/2012) a separate informed consent from individual patients was waived.

### Determination of IDH1/IDH2, ATRX, p53, and 1p/19q status

Immunohistochemistry for IDH1, ATRX, and p53 mutation were performed using tissue microarray (TMA) blocks sectioned at 4 μm for immunostaining. To build TMA blocks, annotations with a diameter of 1.5 mm were made to the most representative tumor areas in scanned hematoxylin and eosin (H&E) slides using Pannoramic Viewer software (3DHistech, Budapest, Hungary). Corresponding tissue cores from formalin-fixed paraffin-embedded glioma samples were then automatically transferred into TMA blocks using TMA Grand Master (3DHistech, Budapest, Hungary). Stainings were performed on a Ventana Benchmark XT Autostainer (Ventana, Strasbourg, France). Anti-IDH1 R132H antibody at 1:50 dilution (clone H09, Dianova, Hamburg, Germany) was used to detect the most common *IDH1* mutation. ATRX was detected using a rabbit polyclonal antibody at 1:500 dilution (Sigma-Aldrich, St Louis, MO; cat# HPA001906). Loss of nuclear staining in tumor cells while remaining positive staining in non-neoplastic cells indicated *ATRX* mutation. Monoclonal antibody Bp53-11 (Ventana Medical Systems, Tucson, AZ) was used to detect *p53* mutation. Nuclear staining in > 10% of neoplastic cells was regarded as positive for *TP53* mutation. 1p/19q codeletion was studied by fluorescent *in situ* hybridization using Vysis 1p36/1q25 and 19q13/19p13 FISH probe kit (Abbot Laboratories, Abbot Park, IL) in the diagnostic samples.

Samples with negative or failed IDH1 immunohistochemistry were subjected to direct Sanger sequencing. DNA was extracted from cylindrical paraffin-embedded tissue samples and PCR amplification products were disposed to sequencing described by Hartmann et al. [32]. Sequencing was performed in forward direction at Eurofins Genomics (Ebersberg, Germany) using ABI3730XL sequencer (ThermoFischer Scientific, MA, USA). Single ambiguous sequences after repetition were grouped in the NOS category. The sequences were analyzed using Sequencer™ 5.1 software. Codons 132 and 172 were examined to determine the mutation status of *IDH1* and *IDH2* genes, respectively, as previously described [32]. With all the molecular data available, gliomas were re-assessed by an experienced neuropathologist (M.G.) and diagnosed according to the new integrated WHO classification [5].

### SSTR2A immunohistochemistry

Formalin-fixed paraffin-embedded tumor tissues were sectioned at 4 μm and used for SSTR2A immunohistochemistry (monoclonal UMB-1, Abcam, Cambridge, UK; dilution 1:500). To exclude microglia and macrophages from the evaluation, double staining with UMB-1 and CD68 (clone PG-M1, Dako, Glostrup, Denmark; dilution 1:100) was performed as described in [16]. In order to detect the heterogeneous staining pattern of SSTR2A as previously noticed by our group in high-grade gliomas, the double stainings were performed on whole paraffin tissue sections and not TMAs. The chromogen used for CD68 detection was red, while SSTR2A staining was brown. Staining intensity for SSTR2A reaction was reported as 0 (negative), 1 (weak), 2 (moderate), or 3 (strong). Due to heterogeneous staining we evaluated both the most common staining intensity (minimum 50% of tumor area) and the highest staining intensity (minimum 10% of tumor area). Additionally, the localization of the staining (cytoplasmic or both membranous and cytoplasmic) was observed. Scoring of SSTR2A immunohistochemistry was independently performed by a neuropathologist (M.G.) and a research fellow (A.K.). In case of discrepancy, a consensus in the scoring was obtained. SSTR2A status was regarded positive if the most common staining intensity was 2 or 3, or the highest staining intensity was 3.

### Statistics

The association between SSTR2A expression and glioma subtype, *IDH* mutation, and *ATRX* mutation was analyzed using Fisher's exact test. Kaplan-Meier with log-rank test and multivariate Cox proportional hazards regression were performed to assess survival data. OS was defined as the time from surgical resection to death or end of follow-up. PFS was a composite end-point defined as the time from surgical resection to the first tumor progression indicated by re-resection, start of a new treatment regimen, death, or end of follow-up. NOS designated diagnoses were not included in the final analyses. Two-tailed *p*-values < 0.05 were regarded significant. Analyses were conducted using IBM SPSS Statistics version 24.0 (IBM Corp., Armonk, NY, USA).
